# Supplementary material for: Inflammatory Immune Responses in the Pathogenesis of Tick-Borne Encephalitis
Source: J Clin Med. 2019 May 22;8(5):731. doi: 10.3390/jcm8050731 (PMC6571551; doi:10.3390/jcm8050731)
Supplement: Supplementary file 1 [file jcm-08-00731-s001.pdf]

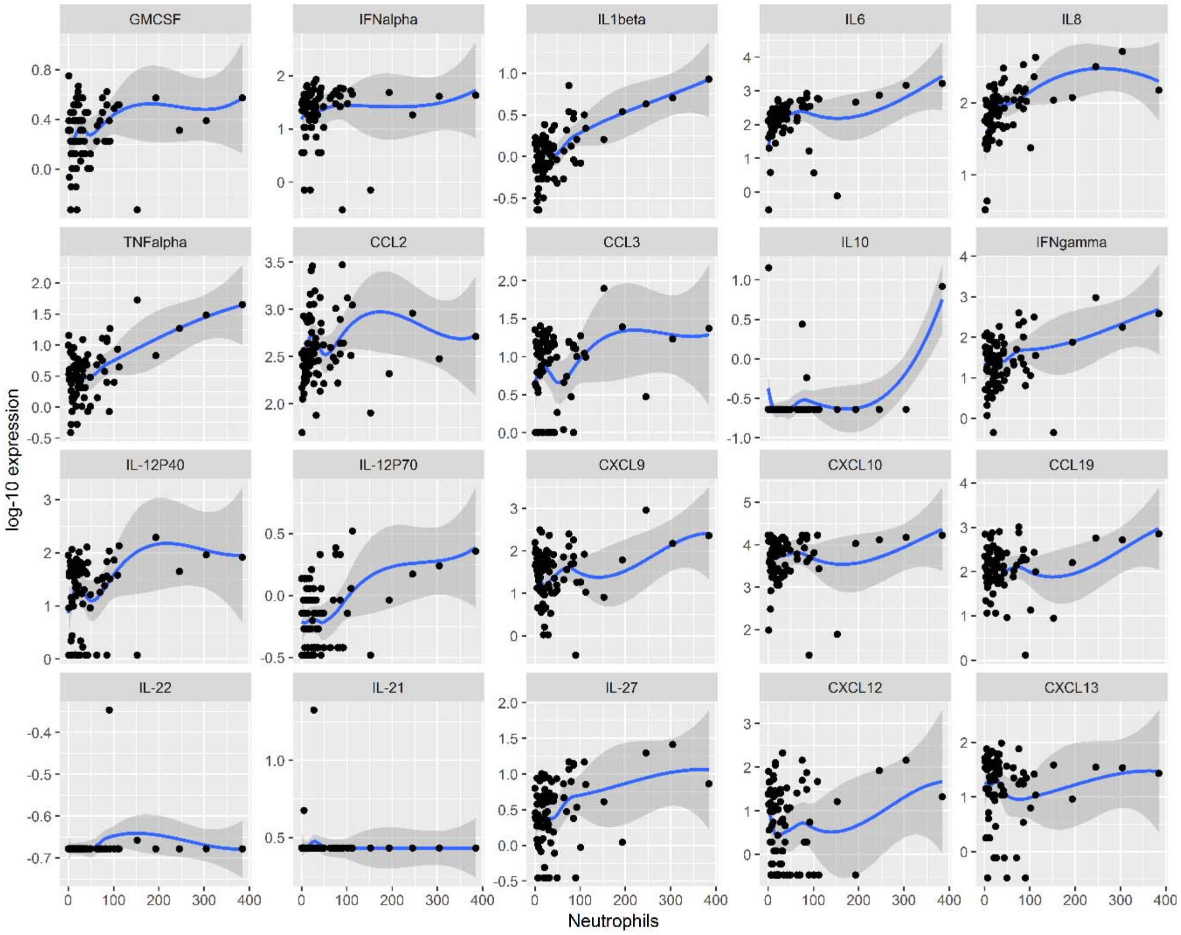

Figure S1. Correlation of Cytokine and Chemokine Levels and Neutrophil Counts in CSF (Determined in the Meningoencephalic Phase of Tick-borne Encephalitis).

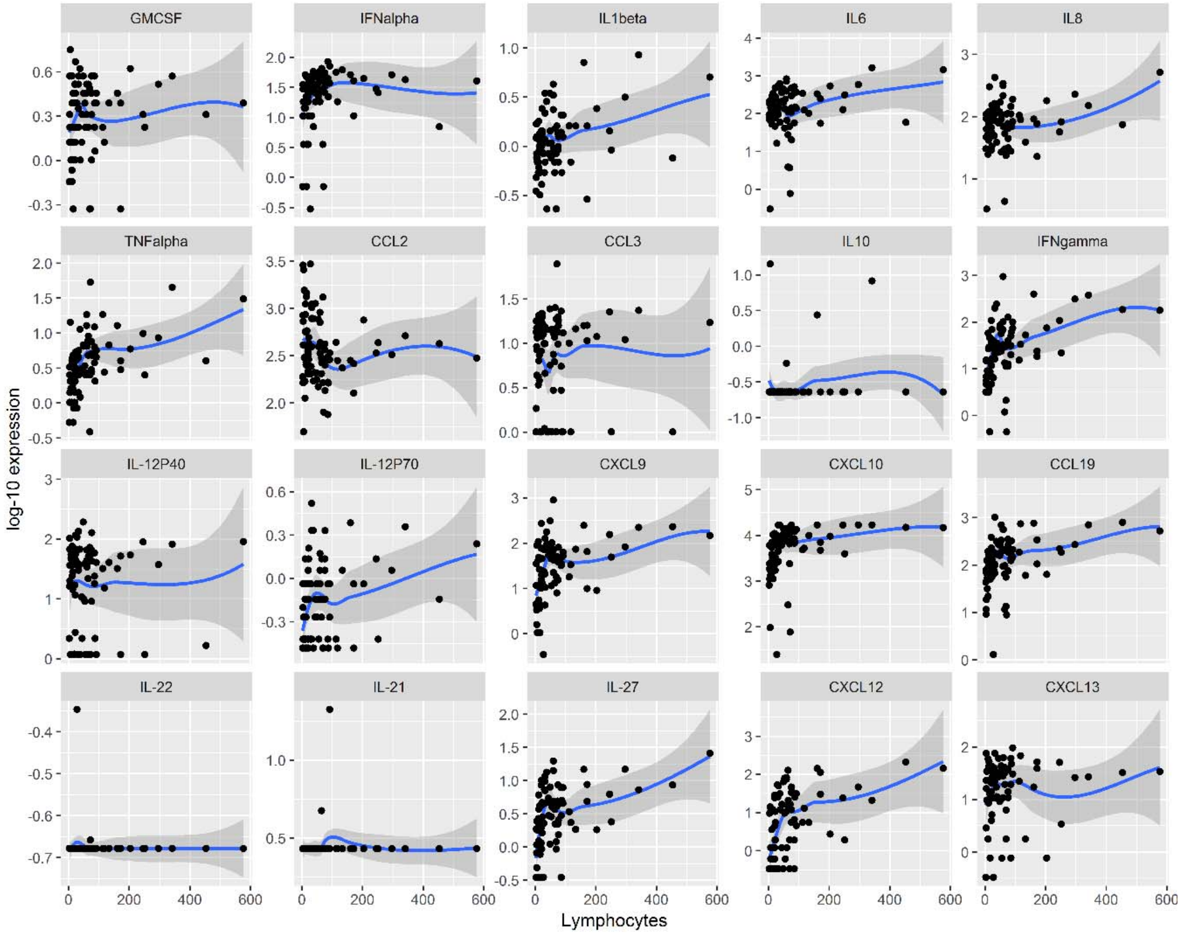

Figure S2. Correlation of Cytokine and Chemokine Levels and Lymphocyte Counts in CSF (Determined in the Meningoencephalic Phase of Tick-borne Encephalitis).

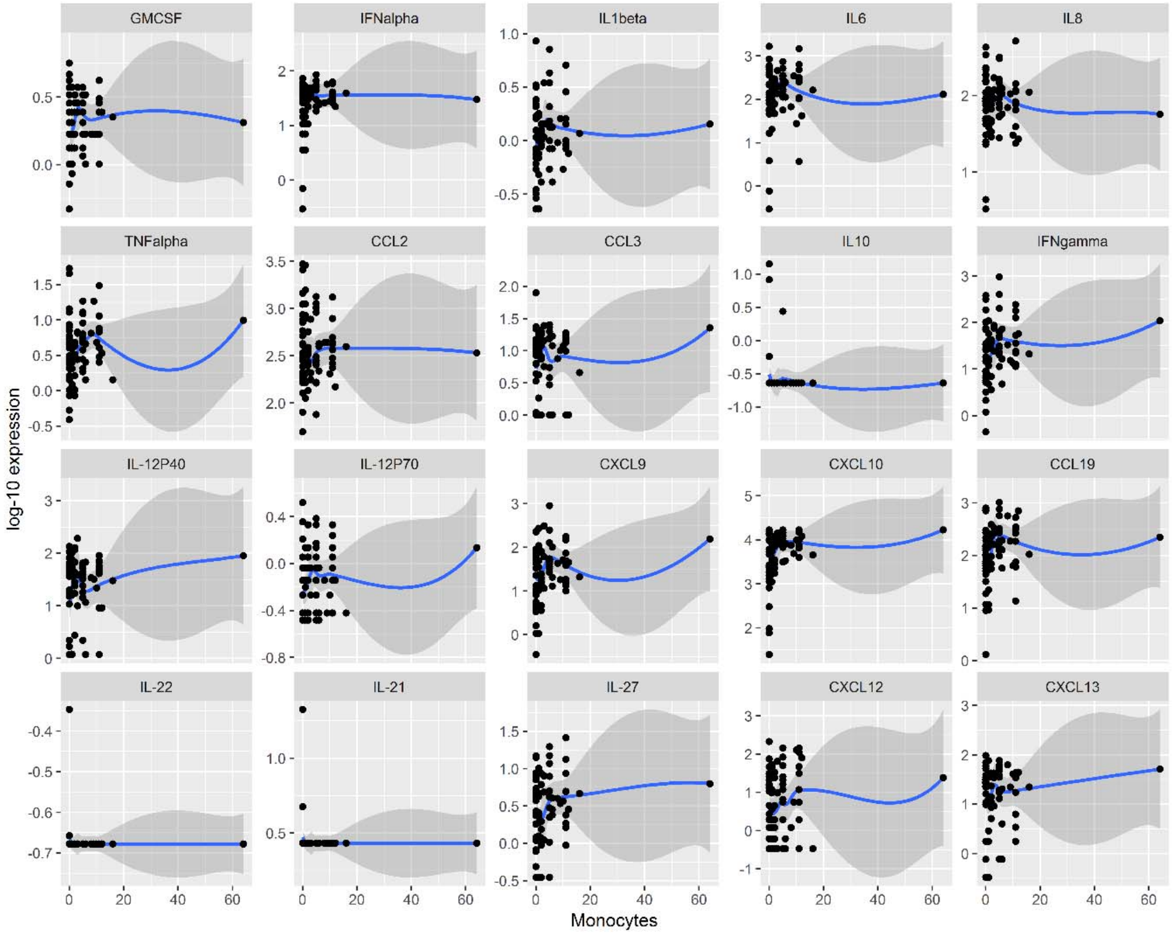

Figure S3. Correlation of Cytokine and Chemokine Levels and Monocyte Counts in CSF (Determined in the Meningoencephalic Phase of Tick-borne Encephalitis).

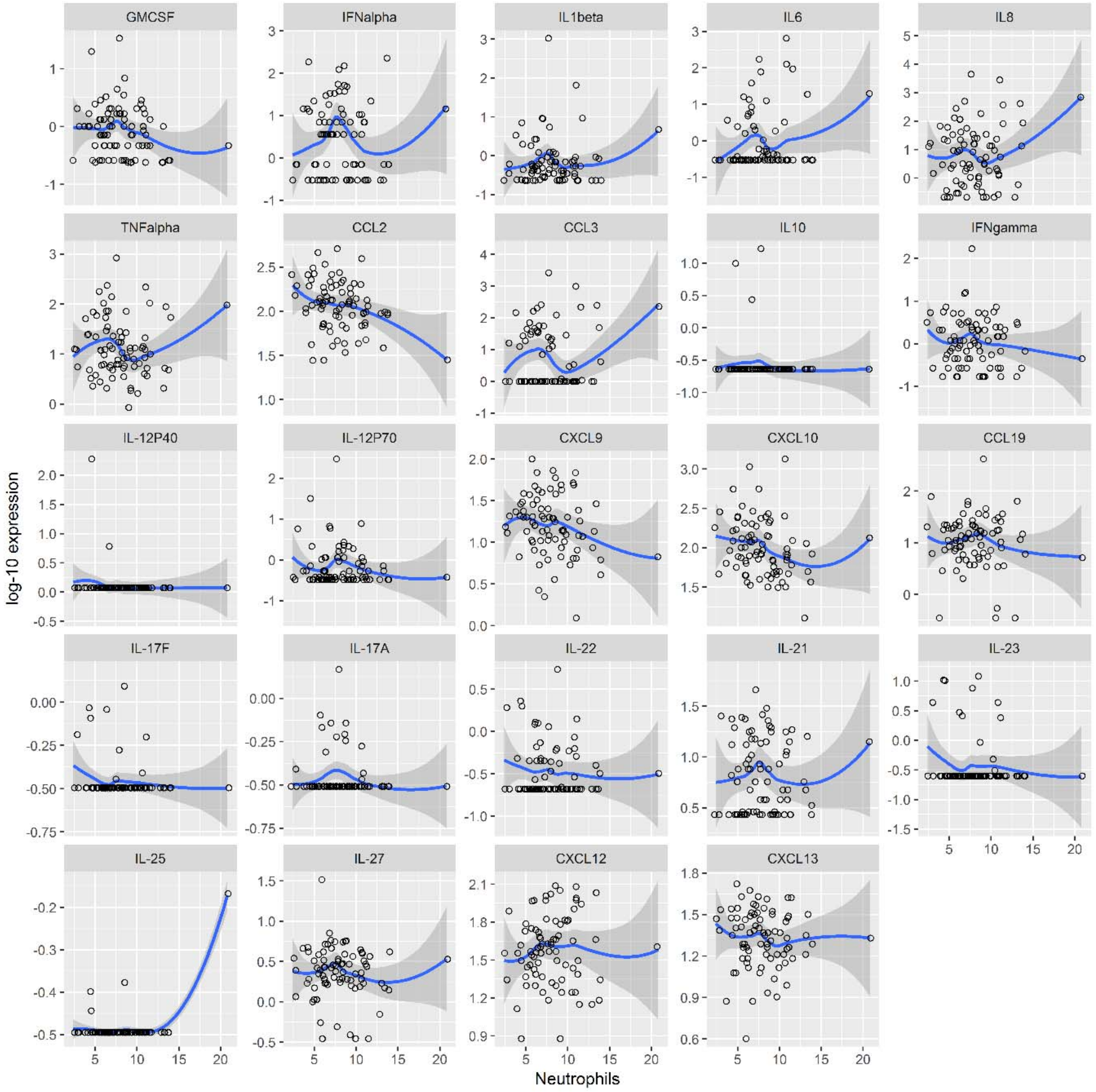

Figure S4. Correlation of Cytokine and Chemokine Levels and Neutrophil Counts in Serum (Determined in the Meningoencephalic Phase of Tick-borne Encephalitis).

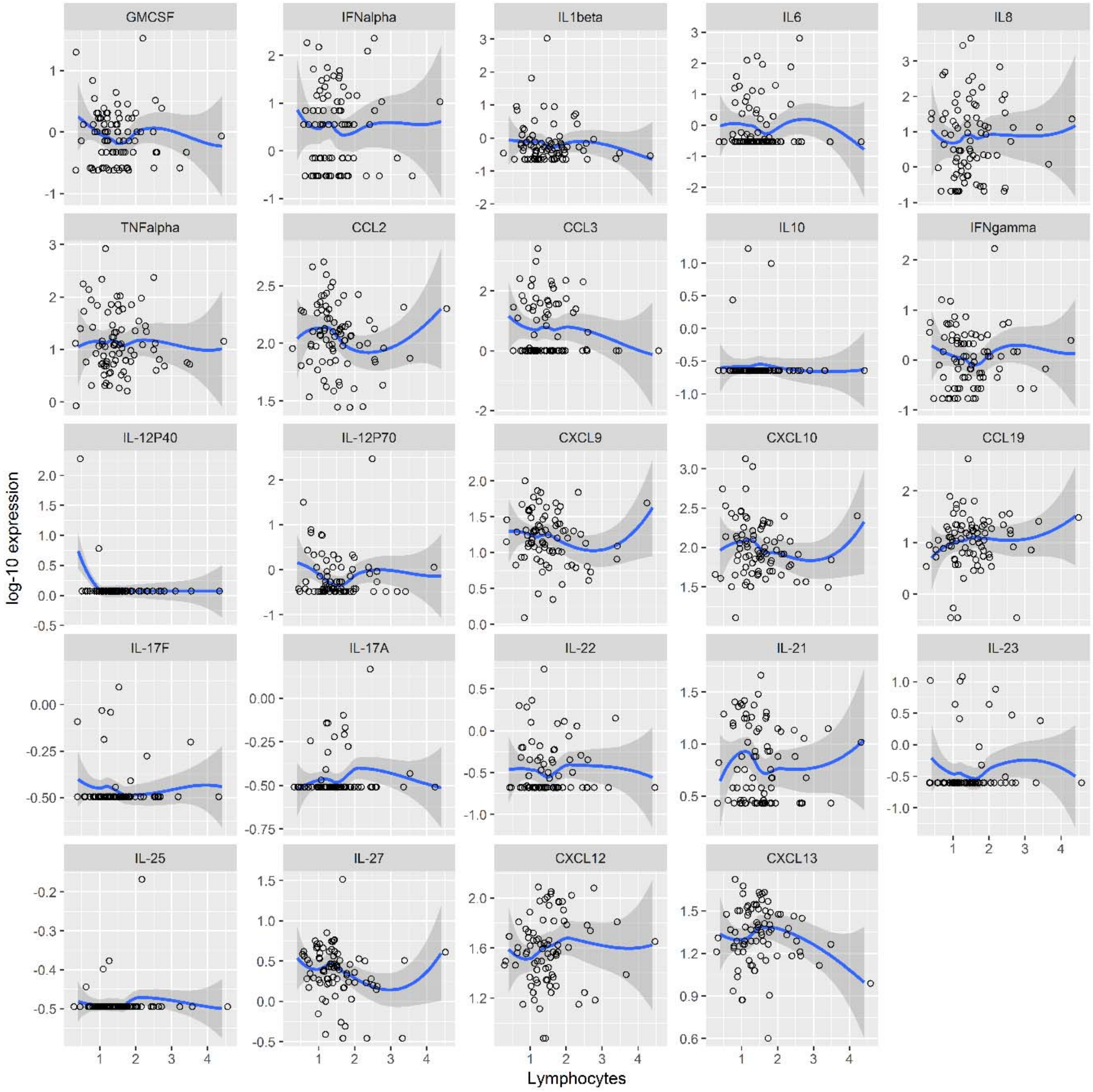

Figure S5. Correlation of Cytokine and Chemokine Levels and Lymphocyte Counts in Serum (Determined in the Meningoencephalic Phase of Tick-borne Encephalitis).

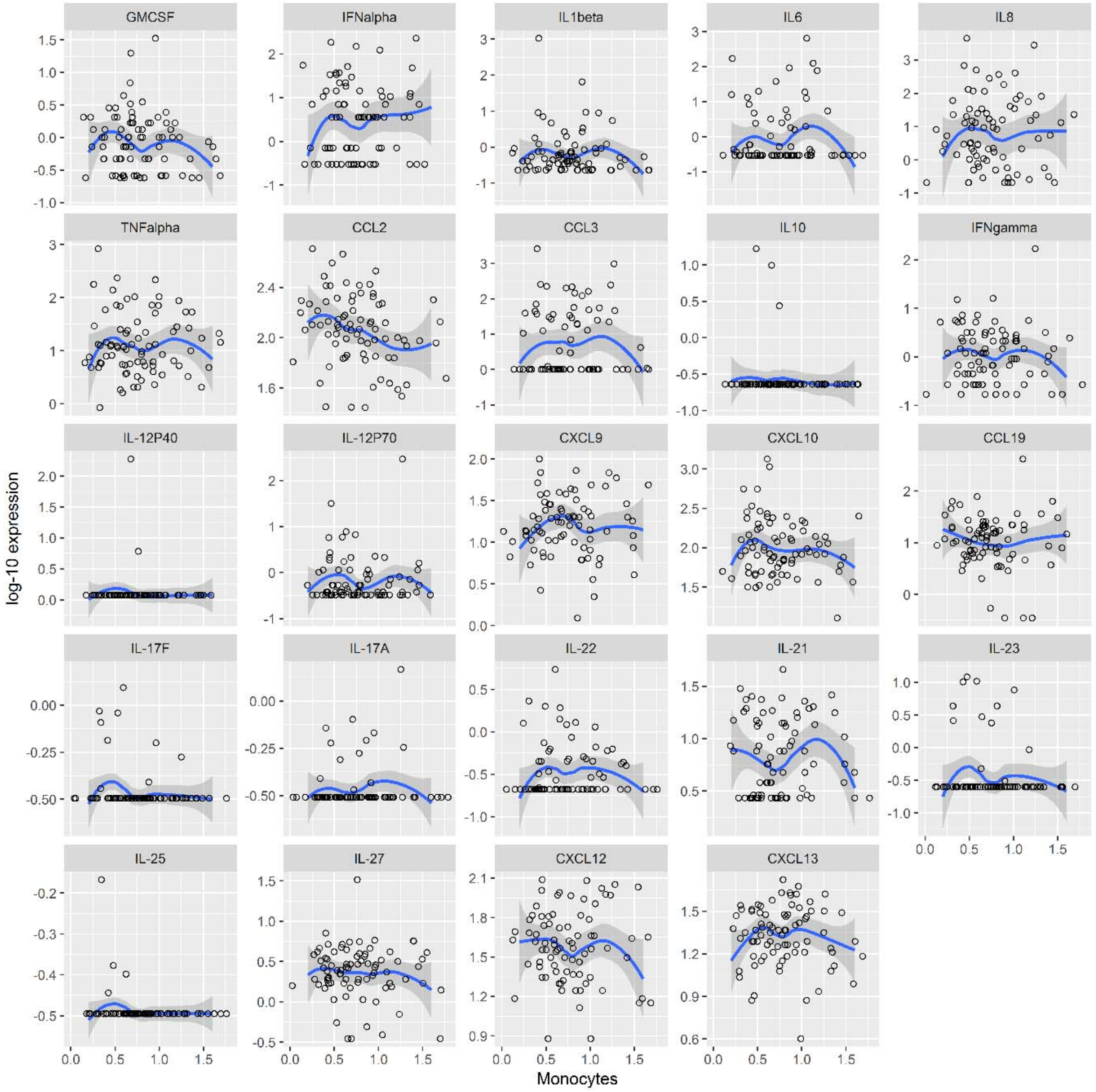

Figure S6. Correlation of Cytokine and Chemokine Levels and Monocyte Counts in Serum (Determined in the Meningoencephalic Phase of Tick-borne Encephalitis).

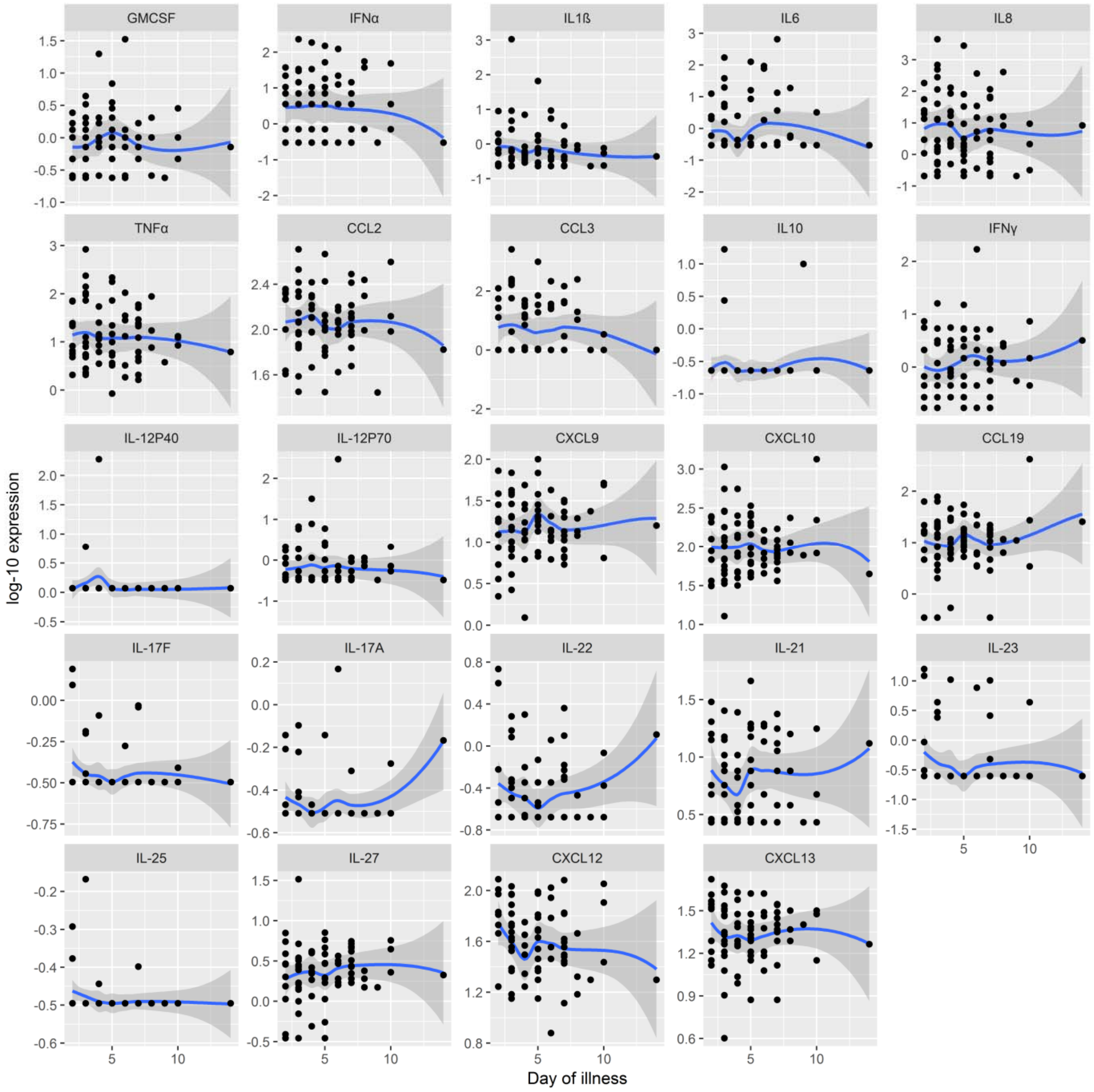

Figure S7. Serum Cytokines and Chemokines Levels according to the Duration (days) of the Meningoencephalitic Phase of Tick-borne Encephalitis.

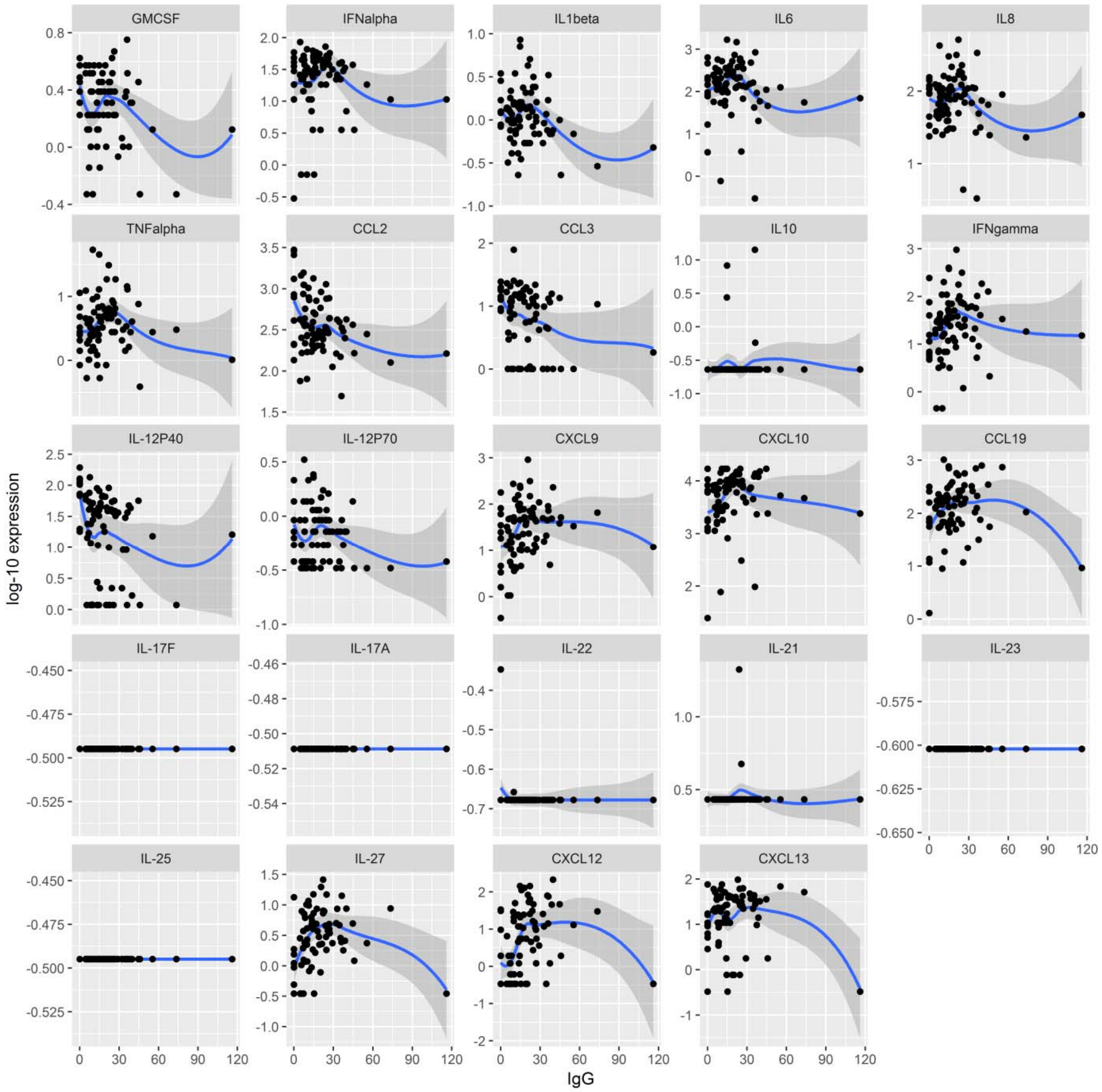

Figure S8. Association of Inflammatory Mediators with Levels of IgG Antibodies Against Tick-borne Encephalitis Virus in Cerebrospinal Fluid.

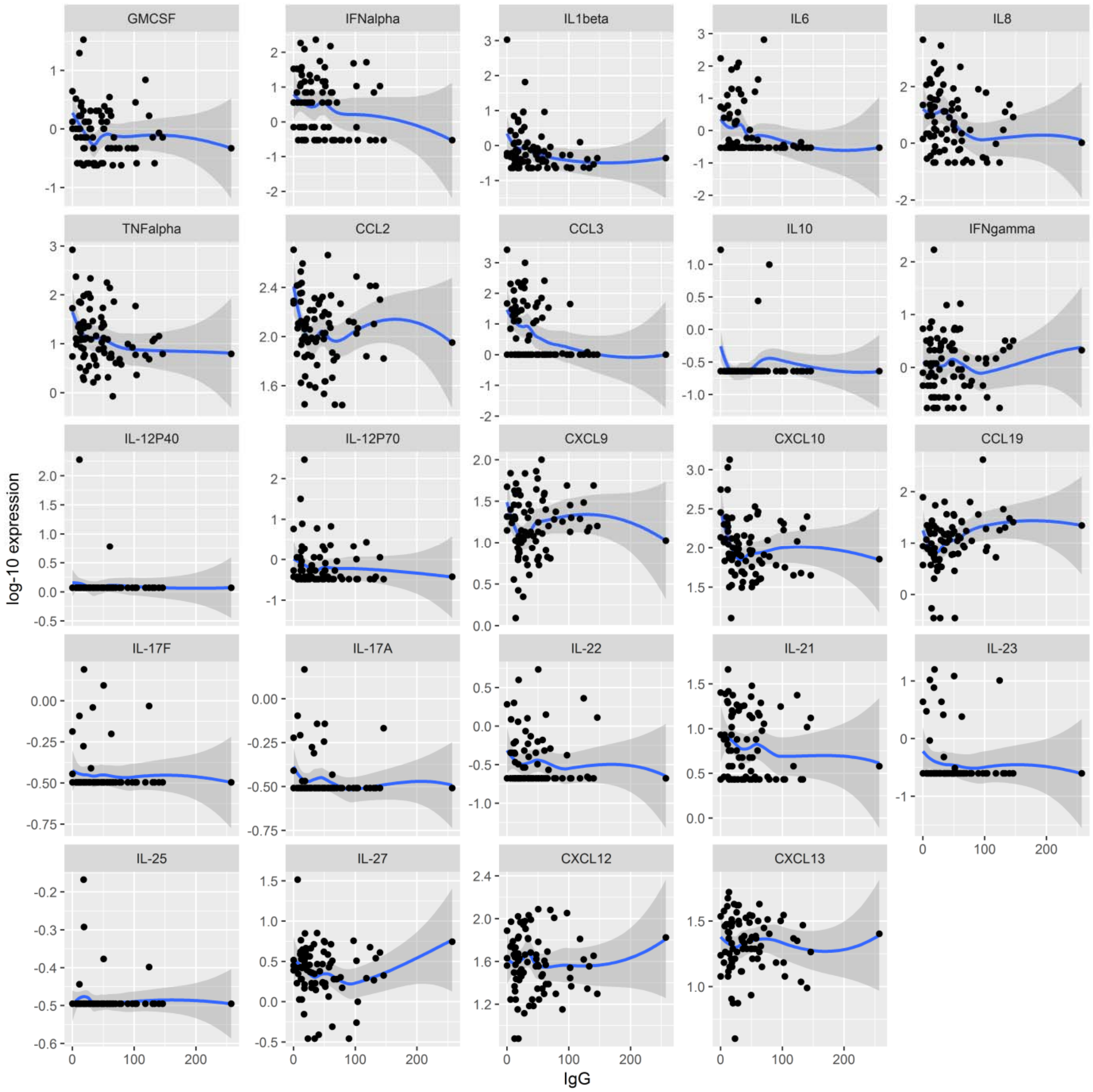

Figure S9. Association of inflammatory mediators with levels of IgG antibodies against Tick-borne Encephalitis Virus in Serum.
